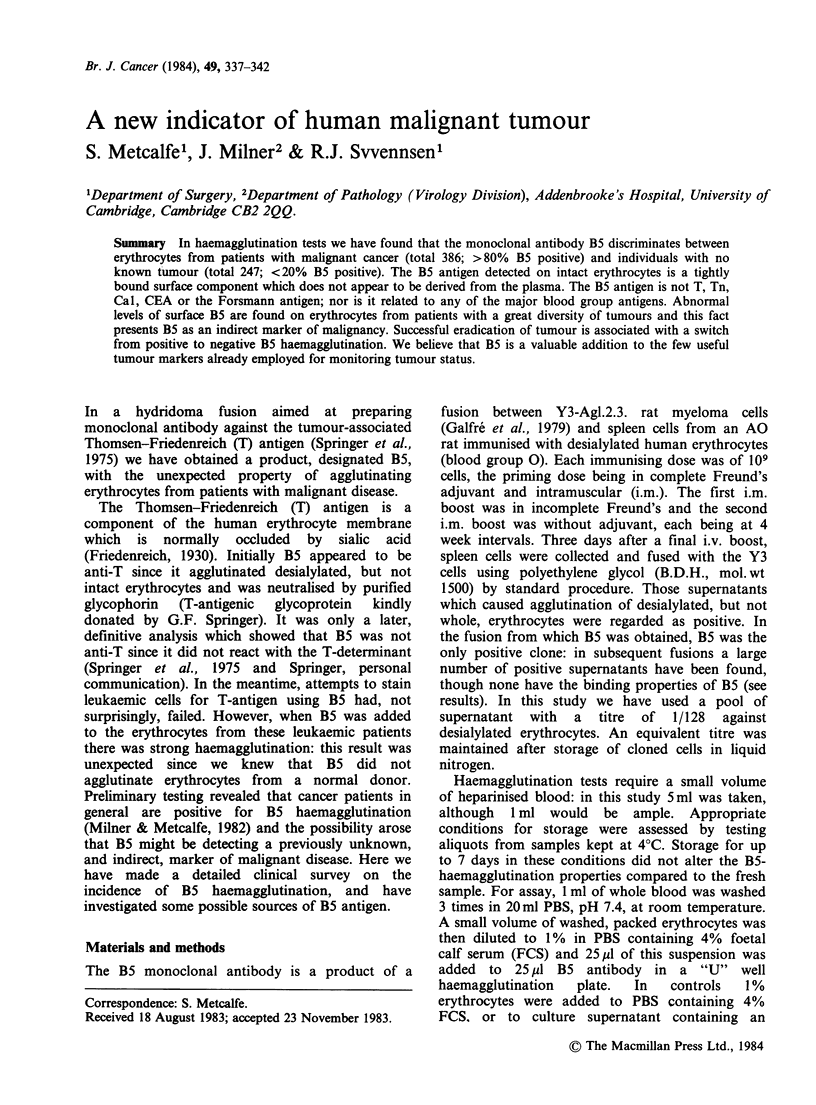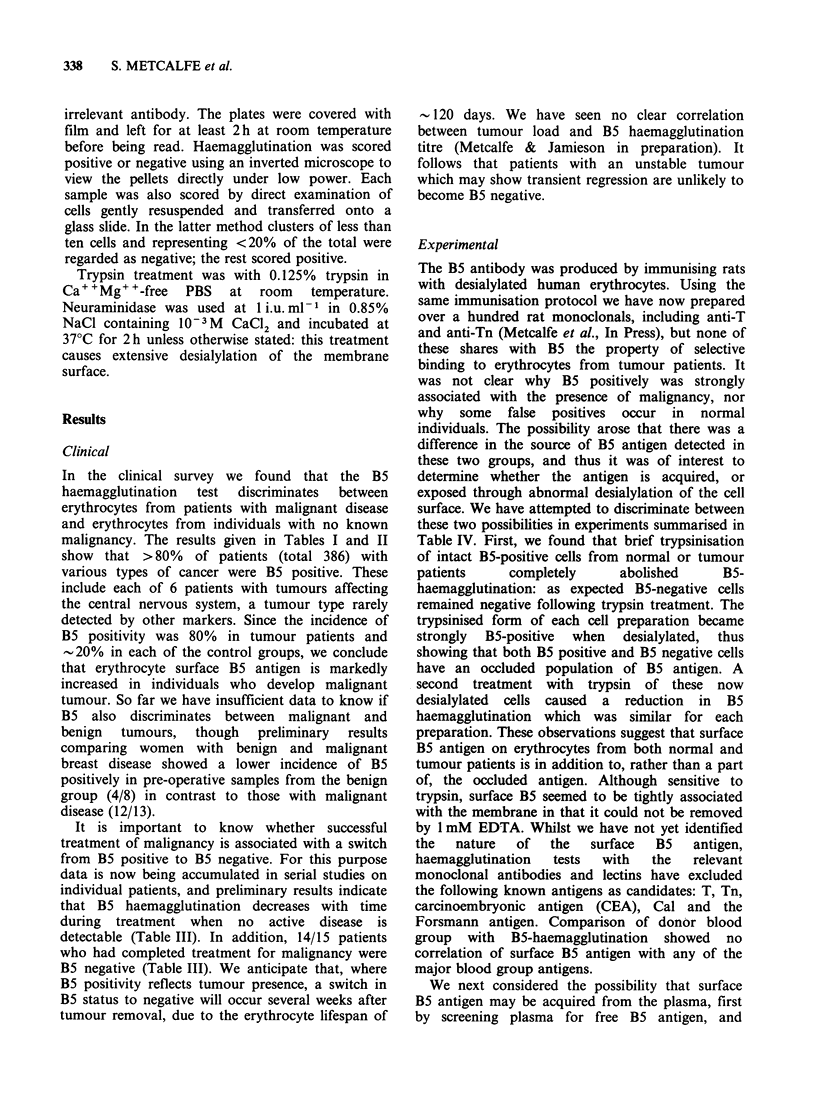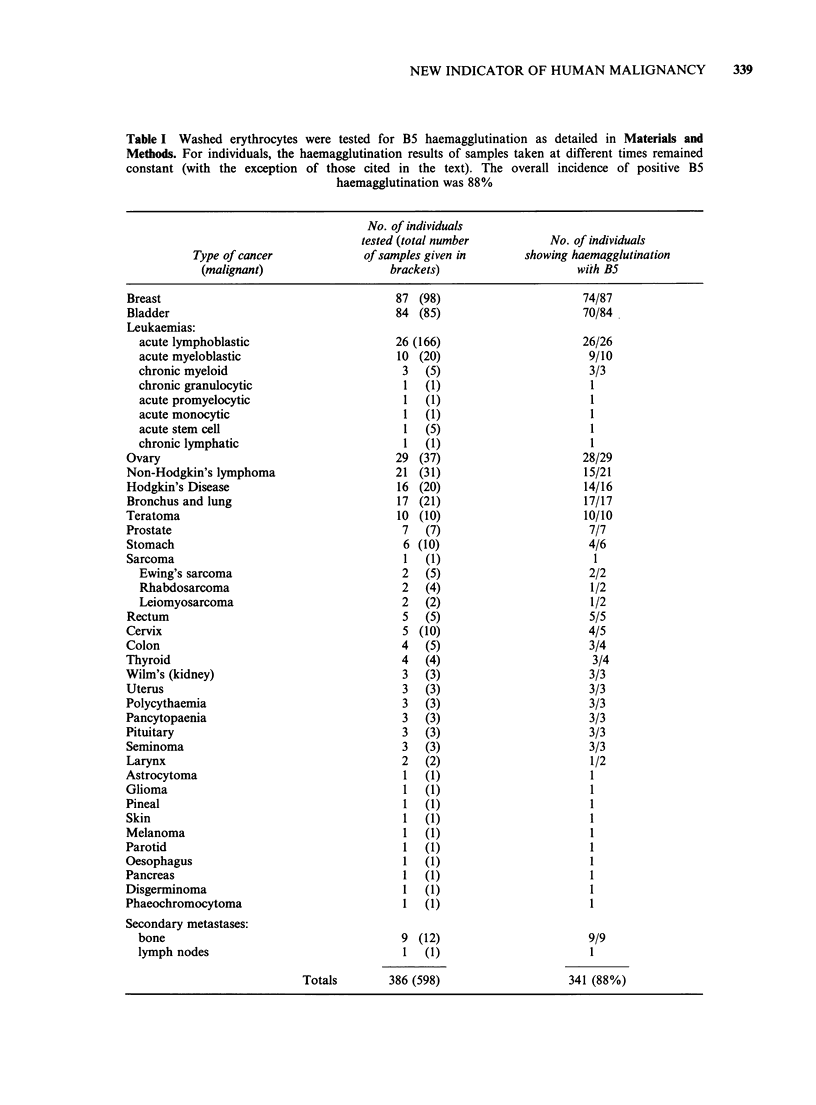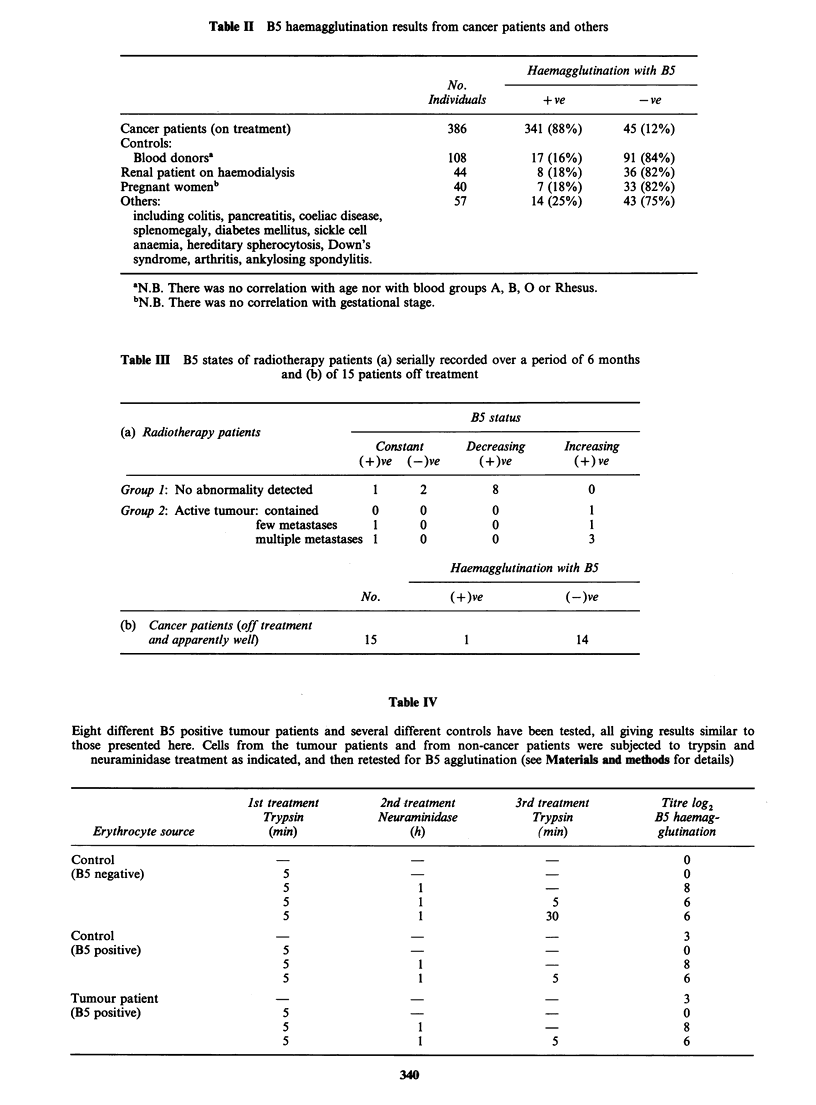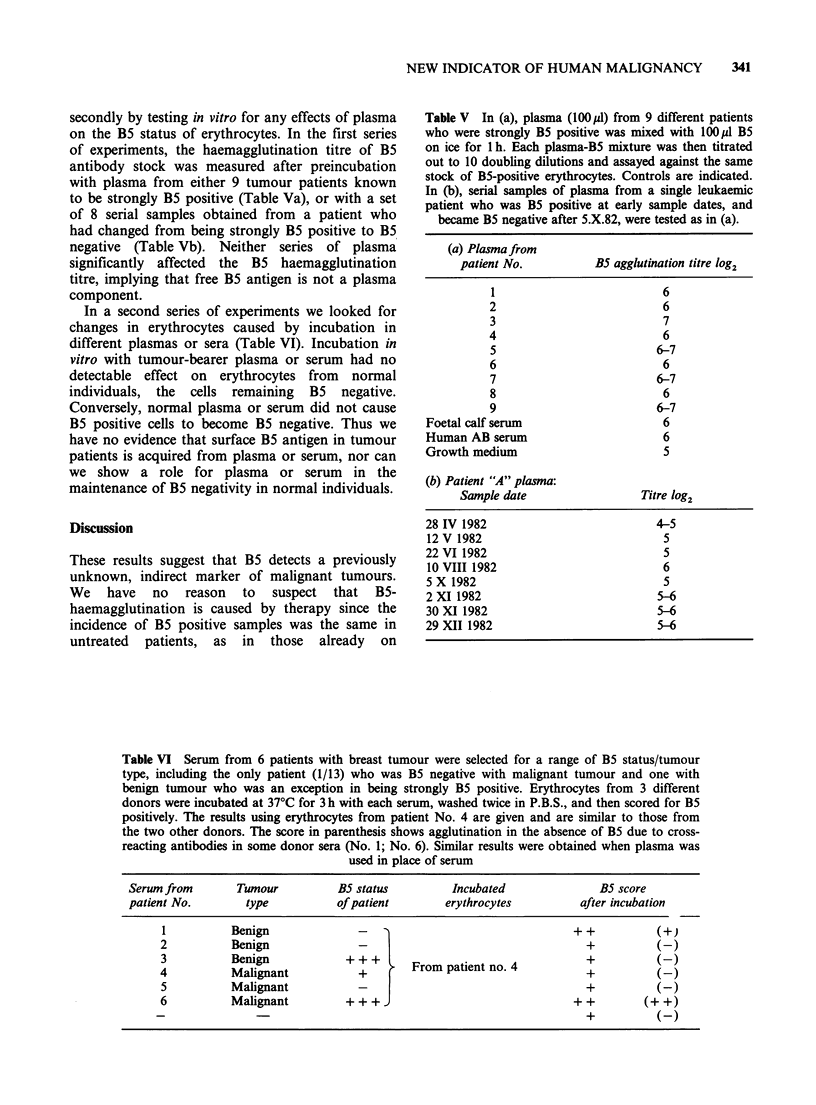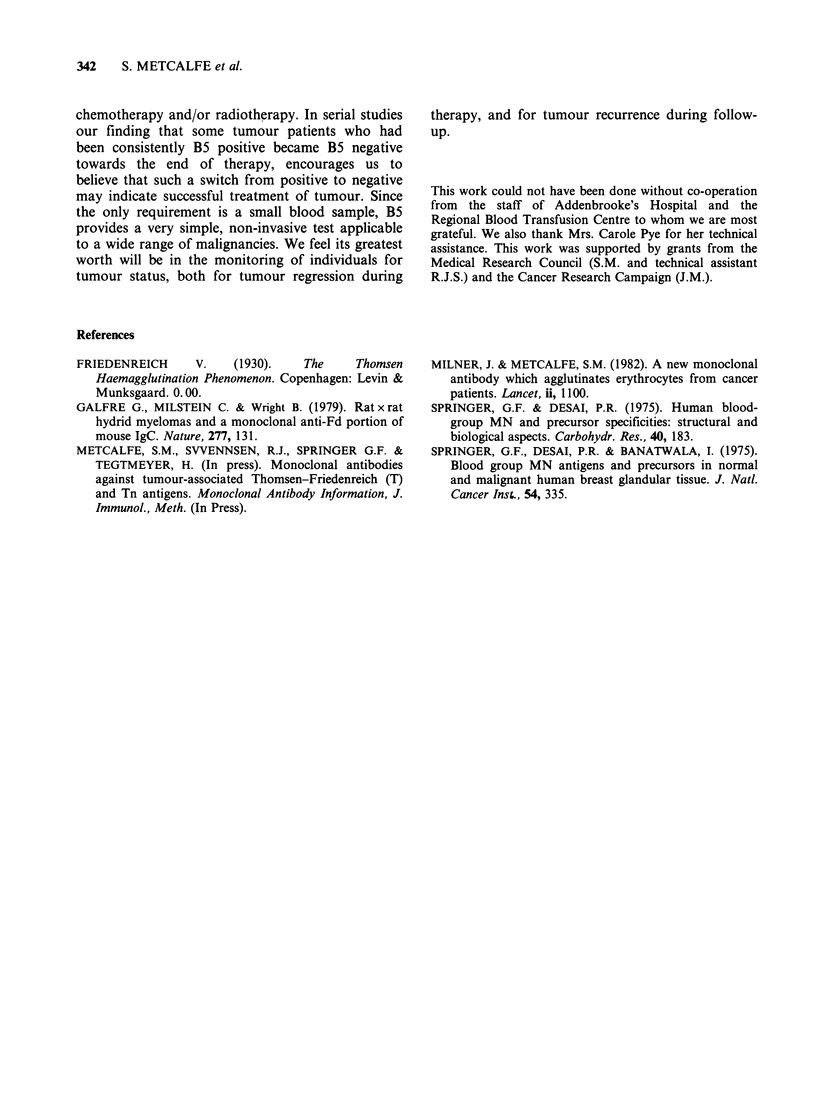# A new indicator of human malignant tumour.

**DOI:** 10.1038/bjc.1984.53

**Published:** 1984-03

**Authors:** S. Metcalfe, J. Milner, R. J. Svvennsen

## Abstract

In haemagglutination tests we have found that the monoclonal antibody B5 discriminates between erythrocytes from patients with malignant cancer (total 386; greater than 80% B5 positive) and individuals with no known tumour (total 247; less than 20% B5 positive). The B5 antigen detected on intact erythrocytes is a tightly bound surface component which does not appear to be derived from the plasma. The B5 antigen is not T, Tn, Ca1, CEA or the Forsmann antigen; nor is it related to any of the major blood group antigens. Abnormal levels of surface B5 are found on erythrocytes from patients with a great diversity of tumours and this fact presents B5 as an indirect marker of malignancy. Successful eradication of tumour is associated with a switch from positive to negative B5 haemagglutination. We believe that B5 is a valuable addition to the few useful tumour markers already employed for monitoring tumour status.


*Br. J. Cancer* (1984), **49**, 337–342

### A new indicator of human malignant tumour

S. Metcalfe[1], J. Milner[2] & R.J. Svvennsen[1]

[1]*Department of Surgery,* [2]*Department of Pathology (Virology Division), Addenbrooke's Hospital, University of Cambridge, Cambridge CB2 2QQ.*

**Summary** In haemagglutination tests we have found that the monoclonal antibody B5 discriminates between erythrocytes from patients with malignant cancer (total 386; >80% B5 positive) and individuals with no known tumour (total 247; <20% B5 positive). The B5 antigen detected on intact erythrocytes is a tightly bound surface component which does not appear to be derived from the plasma. The B5 antigen is not T, Tn, Ca1, CEA or the Forsmann antigen; nor is it related to any of the major blood group antigens. Abnormal levels of surface B5 are found on erythrocytes from patients with a great diversity of tumours and this fact presents B5 as an indirect marker of malignancy. Successful eradication of tumour is associated with a switch from positive to negative B5 haemagglutination. We believe that B5 is a valuable addition to the few useful tumour markers already employed for monitoring tumour status.

In a hydridoma fusion aimed at preparing monoclonal antibody against the tumour-associated Thomsen–Friedenreich (T) antigen (Springer *et al.*, 1975) we have obtained a product, designated B5, with the unexpected property of agglutinating erythrocytes from patients with malignant disease.

The Thomsen–Friedenreich (T) antigen is a component of the human erythrocyte membrane which is normally occluded by sialic acid (Friedenreich, 1930). Initially B5 appeared to be anti-T since it agglutinated desialylated, but not intact erythrocytes and was neutralised by purified glycophorin (T-antigenic glycoprotein kindly donated by G.F. Springer). It was only a later, definitive analysis which showed that B5 was not anti-T since it did not react with the T-determinant (Springer *et al.*, 1975 and Springer, personal communication). In the meantime, attempts to stain leukaemic cells for T-antigen using B5 had, not surprisingly, failed. However, when B5 was added to the erythrocytes from these leukaemic patients there was strong haemagglutination: this result was unexpected since we knew that B5 did not agglutinate erythrocytes from a normal donor. Preliminary testing revealed that cancer patients in general are positive for B5 haemagglutination (Milner & Metcalfe, 1982) and the possibility arose that B5 might be detecting a previously unknown, and indirect, marker of malignant disease. Here we have made a detailed clinical survey on the incidence of B5 haemagglutination, and have investigated some possible sources of B5 antigen.

### Materials and methods

The B5 monoclonal antibody is a product of a fusion between Y3-Agl.2.3. rat myeloma cells (Galfré *et al.*, 1979) and spleen cells from an AO rat immunised with desialylated human erythrocytes (blood group O). Each immunising dose was of $10^9$ cells, the priming dose being in complete Freund's adjuvant and intramuscular (i.m.). The first i.m. boost was in incomplete Freund's and the second i.m. boost was without adjuvant, each being at 4 week intervals. Three days after a final i.v. boost, spleen cells were collected and fused with the Y3 cells using polyethylene glycol (B.D.H., mol. wt 1500) by standard procedure. Those supernatants which caused agglutination of desialylated, but not whole, erythrocytes were regarded as positive. In the fusion from which B5 was obtained, B5 was the only positive clone: in subsequent fusions a large number of positive supernatants have been found, though none have the binding properties of B5 (see results). In this study we have used a pool of supernatant with a titre of 1/128 against desialylated erythrocytes. An equivalent titre was maintained after storage of cloned cells in liquid nitrogen.

Haemagglutination tests require a small volume of heparinised blood: in this study 5 ml was taken, although 1 ml would be ample. Appropriate conditions for storage were assessed by testing aliquots from samples kept at 4°C. Storage for up to 7 days in these conditions did not alter the B5-haemagglutination properties compared to the fresh sample. For assay, 1 ml of whole blood was washed 3 times in 20 ml PBS, pH 7.4, at room temperature. A small volume of washed, packed erythrocytes was then diluted to 1% in PBS containing 4% foetal calf serum (FCS) and 25 $\mu$l of this suspension was added to 25 $\mu$l B5 antibody in a "U" well haemagglutination plate. In controls 1% erythrocytes were added to PBS containing 4% FCS, or to culture supernatant containing an

Correspondence: S. Metcalfe.
Received 18 August 1983; accepted 23 November 1983.

© The Macmillan Press Ltd., 1984

irrelevant antibody. The plates were covered with film and left for at least 2 h at room temperature before being read. Haemagglutination was scored positive or negative using an inverted microscope to view the pellets directly under low power. Each sample was also scored by direct examination of cells gently resuspended and transferred onto a glass slide. In the latter method clusters of less than ten cells and representing <20% of the total were regarded as negative; the rest scored positive.

Trypsin treatment was with 0.125% trypsin in $Ca^{++}Mg^{++}$-free PBS at room temperature. Neuraminidase was used at 1 i.u. $ml^{-1}$ in 0.85% NaCl containing $10^{-3}$ M $CaCl_2$ and incubated at 37°C for 2 h unless otherwise stated: this treatment causes extensive desialylation of the membrane surface.

### Results

### *Clinical*

In the clinical survey we found that the B5 haemagglutination test discriminates between erythrocytes from patients with malignant disease and erythrocytes from individuals with no known malignancy. The results given in Tables I and II show that >80% of patients (total 386) with various types of cancer were B5 positive. These include each of 6 patients with tumours affecting the central nervous system, a tumour type rarely detected by other markers. Since the incidence of B5 positivity was 80% in tumour patients and ~20% in each of the control groups, we conclude that erythrocyte surface B5 antigen is markedly increased in individuals who develop malignant tumour. So far we have insufficient data to know if B5 also discriminates between malignant and benign tumours, though preliminary results comparing women with benign and malignant breast disease showed a lower incidence of B5 positively in pre-operative samples from the benign group (4/8) in contrast to those with malignant disease (12/13).

It is important to know whether successful treatment of malignancy is associated with a switch from B5 positive to B5 negative. For this purpose data is now being accumulated in serial studies on individual patients, and preliminary results indicate that B5 haemagglutination decreases with time during treatment when no active disease is detectable (Table III). In addition, 14/15 patients who had completed treatment for malignancy were B5 negative (Table III). We anticipate that, where B5 positivity reflects tumour presence, a switch in B5 status to negative will occur several weeks after tumour removal, due to the erythrocyte lifespan of ~120 days. We have seen no clear correlation between tumour load and B5 haemagglutination titre (Metcalfe & Jamieson in preparation). It follows that patients with an unstable tumour which may show transient regression are unlikely to become B5 negative.

### *Experimental*

The B5 antibody was produced by immunising rats with desialylated human erythrocytes. Using the same immunisation protocol we have now prepared over a hundred rat monoclonals, including anti-T and anti-Tn (Metcalfe *et al*., In Press), but none of these shares with B5 the property of selective binding to erythrocytes from tumour patients. It was not clear why B5 positively was strongly associated with the presence of malignancy, nor why some false positives occur in normal individuals. The possibility arose that there was a difference in the source of B5 antigen detected in these two groups, and thus it was of interest to determine whether the antigen is acquired, or exposed through abnormal desialylation of the cell surface. We have attempted to discriminate between these two possibilities in experiments summarised in Table IV. First, we found that brief trypsinisation of intact B5-positive cells from normal or tumour patients completely abolished B5-haemagglutination: as expected B5-negative cells remained negative following trypsin treatment. The trypsinised form of each cell preparation became strongly B5-positive when desialylated, thus showing that both B5 positive and B5 negative cells have an occluded population of B5 antigen. A second treatment with trypsin of these now desialylated cells caused a reduction in B5 haemagglutination which was similar for each preparation. These observations suggest that surface B5 antigen on erythrocytes from both normal and tumour patients is in addition to, rather than a part of, the occluded antigen. Although sensitive to trypsin, surface B5 seemed to be tightly associated with the membrane in that it could not be removed by 1 mM EDTA. Whilst we have not yet identified the nature of the surface B5 antigen, haemagglutination tests with the relevant monoclonal antibodies and lectins have excluded the following known antigens as candidates: T, Tn, carcinoembryonic antigen (CEA), Cal and the Forsmann antigen. Comparison of donor blood group with B5-haemagglutination showed no correlation of surface B5 antigen with any of the major blood group antigens.

We next considered the possibility that surface B5 antigen may be acquired from the plasma, first by screening plasma for free B5 antigen, and

**Table I** Washed erythrocytes were tested for B5 haemagglutination as detailed in **Materials and Methods.** For individuals, the haemagglutination results of samples taken at different times remained constant (with the exception of those cited in the text). The overall incidence of positive B5 haemagglutination was 88%

| *Type of cancer (malignant)* | *No. of individuals tested (total number of samples given in brackets)* | *No. of individuals showing haemagglutination with B5* |
|---|---|---|
| Breast | 87 (98) | 74/87 |
| Bladder | 84 (85) | 70/84 |
| Leukaemias: | | |
| acute lymphoblastic | 26 (166) | 26/26 |
| acute myeloblastic | 10 (20) | 9/10 |
| chronic myeloid | 3 (5) | 3/3 |
| chronic granulocytic | 1 (1) | 1 |
| acute promyelocytic | 1 (1) | 1 |
| acute monocytic | 1 (1) | 1 |
| acute stem cell | 1 (5) | 1 |
| chronic lymphatic | 1 (1) | 1 |
| Ovary | 29 (37) | 28/29 |
| Non-Hodgkin's lymphoma | 21 (31) | 15/21 |
| Hodgkin's Disease | 16 (20) | 14/16 |
| Bronchus and lung | 17 (21) | 17/17 |
| Teratoma | 10 (10) | 10/10 |
| Prostate | 7 (7) | 7/7 |
| Stomach | 6 (10) | 4/6 |
| Sarcoma | 1 (1) | 1 |
| Ewing's sarcoma | 2 (5) | 2/2 |
| Rhabdosarcoma | 2 (4) | 1/2 |
| Leiomyosarcoma | 2 (2) | 1/2 |
| Rectum | 5 (5) | 5/5 |
| Cervix | 5 (10) | 4/5 |
| Colon | 4 (5) | 3/4 |
| Thyroid | 4 (4) | 3/4 |
| Wilm's (kidney) | 3 (3) | 3/3 |
| Uterus | 3 (3) | 3/3 |
| Polycythaemia | 3 (3) | 3/3 |
| Pancytopaenia | 3 (3) | 3/3 |
| Pituitary | 3 (3) | 3/3 |
| Seminoma | 3 (3) | 3/3 |
| Larynx | 2 (2) | 1/2 |
| Astrocytoma | 1 (1) | 1 |
| Glioma | 1 (1) | 1 |
| Pineal | 1 (1) | 1 |
| Skin | 1 (1) | 1 |
| Melanoma | 1 (1) | 1 |
| Parotid | 1 (1) | 1 |
| Oesophagus | 1 (1) | 1 |
| Pancreas | 1 (1) | 1 |
| Disgerminoma | 1 (1) | 1 |
| Phaeochromocytoma | 1 (1) | 1 |
| Secondary metastases: | | |
| bone | 9 (12) | 9/9 |
| lymph nodes | 1 (1) | 1 |
| Totals | 386 (598) | 341 (88%) |

**Table II** B5 haemagglutination results from cancer patients and others

| | No. Individuals | *Haemagglutination with B5* | |
|---|---|---|---|
| | | *+ve* | *−ve* |
| Cancer patients (on treatment) | 386 | 341 (88%) | 45 (12%) |
| Controls: | | | |
| Blood donors[a] | 108 | 17 (16%) | 91 (84%) |
| Renal patient on haemodialysis | 44 | 8 (18%) | 36 (82%) |
| Pregnant women[b] | 40 | 7 (18%) | 33 (82%) |
| Others: including colitis, pancreatitis, coeliac disease, splenomegaly, diabetes mellitus, sickle cell anaemia, hereditary spherocytosis, Down's syndrome, arthritis, ankylosing spondylitis. | 57 | 14 (25%) | 43 (75%) |

[a]N.B. There was no correlation with age nor with blood groups A, B, O or Rhesus.
[b]N.B. There was no correlation with gestational stage.

**Table III** B5 states of radiotherapy patients (a) serially recorded over a period of 6 months and (b) of 15 patients off treatment

| (a) *Radiotherapy patients* | *B5 status* | | | |
|---|---|---|---|---|
| | *Constant* (+)*ve* | *Constant* (−)*ve* | *Decreasing* (+)*ve* | *Increasing* (+)*ve* |
| *Group 1*: No abnormality detected | 1 | 2 | 8 | 0 |
| *Group 2*: Active tumour: contained | 0 | 0 | 0 | 1 |
| few metastases | 1 | 0 | 0 | 1 |
| multiple metastases | 1 | 0 | 0 | 3 |

| | No. | *Haemagglutination with B5* (+)*ve* | *Haemagglutination with B5* (−)*ve* |
|---|---|---|---|
| (b) *Cancer patients (off treatment and apparently well)* | 15 | 1 | 14 |

**Table IV**

Eight different B5 positive tumour patients and several different controls have been tested, all giving results similar to those presented here. Cells from the tumour patients and from non-cancer patients were subjected to trypsin and neuraminidase treatment as indicated, and then retested for B5 agglutination (see **Materials and methods** for details)

| *Erythrocyte source* | *1st treatment Trypsin (min)* | *2nd treatment Neuraminidase (h)* | *3rd treatment Trypsin (min)* | *Titre $log_2$ B5 haemagglutination* |
|---|---|---|---|---|
| Control | — | — | — | 0 |
| (B5 negative) | 5 | — | — | 0 |
| | 5 | 1 | — | 8 |
| | 5 | 1 | 5 | 6 |
| | 5 | 1 | 30 | 6 |
| Control | — | — | — | 3 |
| (B5 positive) | 5 | — | — | 0 |
| | 5 | 1 | — | 8 |
| | 5 | 1 | 5 | 6 |
| Tumour patient | — | — | — | 3 |
| (B5 positive) | 5 | — | — | 0 |
| | 5 | 1 | — | 8 |
| | 5 | 1 | 5 | 6 |

secondly by testing *in vitro* for any effects of plasma on the B5 status of erythrocytes. In the first series of experiments, the haemagglutination titre of B5 antibody stock was measured after preincubation with plasma from either 9 tumour patients known to be strongly B5 positive (Table Va), or with a set of 8 serial samples obtained from a patient who had changed from being strongly B5 positive to B5 negative (Table Vb). Neither series of plasma significantly affected the B5 haemagglutination titre, implying that free B5 antigen is not a plasma component.

In a second series of experiments we looked for changes in erythrocytes caused by incubation in different plasmas or sera (Table VI). Incubation *in vitro* with tumour-bearer plasma or serum had no detectable effect on erythrocytes from normal individuals, the cells remaining B5 negative. Conversely, normal plasma or serum did not cause B5 positive cells to become B5 negative. Thus we have no evidence that surface B5 antigen in tumour patients is acquired from plasma or serum, nor can we show a role for plasma or serum in the maintenance of B5 negativity in normal individuals.

### Discussion

These results suggest that B5 detects a previously unknown, indirect marker of malignant tumours. We have no reason to suspect that B5-haemagglutination is caused by therapy since the incidence of B5 positive samples was the same in untreated patients, as in those already on

**Table V** In (a), plasma (100 μl) from 9 different patients who were strongly B5 positive was mixed with 100 μl B5 on ice for 1 h. Each plasma-B5 mixture was then titrated out to 10 doubling dilutions and assayed against the same stock of B5-positive erythrocytes. Controls are indicated. In (b), serial samples of plasma from a single leukaemic patient who was B5 positive at early sample dates, and became B5 negative after 5.X.82, were tested as in (a).

| (*a*) *Plasma from patient No.* | *B5 agglutination titre* $log_2$ |
|---|---|
| 1 | 6 |
| 2 | 6 |
| 3 | 7 |
| 4 | 6 |
| 5 | 6–7 |
| 6 | 6 |
| 7 | 6–7 |
| 8 | 6 |
| 9 | 6–7 |
| Foetal calf serum | 6 |
| Human AB serum | 6 |
| Growth medium | 5 |

| (*b*) *Patient "A" plasma: Sample date* | *Titre* $log_2$ |
|---|---|
| 28 IV 1982 | 4–5 |
| 12 V 1982 | 5 |
| 22 VI 1982 | 5 |
| 10 VIII 1982 | 6 |
| 5 X 1982 | 5 |
| 2 XI 1982 | 5–6 |
| 30 XI 1982 | 5–6 |
| 29 XII 1982 | 5–6 |

**Table VI** Serum from 6 patients with breast tumour were selected for a range of B5 status/tumour type, including the only patient (1/13) who was B5 negative with malignant tumour and one with benign tumour who was an exception in being strongly B5 positive. Erythrocytes from 3 different donors were incubated at 37°C for 3 h with each serum, washed twice in P.B.S., and then scored for B5 positively. The results using erythrocytes from patient No. 4 are given and are similar to those from the two other donors. The score in parenthesis shows agglutination in the absence of B5 due to cross-reacting antibodies in some donor sera (No. 1; No. 6). Similar results were obtained when plasma was used in place of serum

| *Serum from patient No.* | *Tumour type* | *B5 status of patient* | *Incubated erythrocytes* | *B5 score after incubation* | |
|---|---|---|---|---|---|
| 1 | Benign | – | From patient no. 4 | ++ | (+) |
| 2 | Benign | – | | + | (–) |
| 3 | Benign | +++ | | + | (–) |
| 4 | Malignant | + | | + | (–) |
| 5 | Malignant | – | | + | (–) |
| 6 | Malignant | +++ | | ++ | (++) |
| – | — | | | + | (–) |

chemotherapy and/or radiotherapy. In serial studies our finding that some tumour patients who had been consistently B5 positive became B5 negative towards the end of therapy, encourages us to believe that such a switch from positive to negative may indicate successful treatment of tumour. Since the only requirement is a small blood sample, B5 provides a very simple, non-invasive test applicable to a wide range of malignancies. We feel its greatest worth will be in the monitoring of individuals for tumour status, both for tumour regression during therapy, and for tumour recurrence during follow-up.

This work could not have been done without co-operation from the staff of Addenbrooke's Hospital and the Regional Blood Transfusion Centre to whom we are most grateful. We also thank Mrs. Carole Pye for her technical assistance. This work was supported by grants from the Medical Research Council (S.M. and technical assistant R.J.S.) and the Cancer Research Campaign (J.M.).

### References

FRIEDENREICH V. (1930). *The Thomsen Haemagglutination Phenomenon*. Copenhagen: Levin & Munksgaard. 0.00.

GALFRE G., MILSTEIN C. & Wright B. (1979). Rat x rat hydrid myelomas and a monoclonal anti-Fd portion of mouse IgC. *Nature*, **277**, 131.

METCALFE, S.M., SVVENNSEN, R.J., SPRINGER G.F. & TEGTMEYER, H. (In press). Monoclonal antibodies against tumour-associated Thomsen–Friedenreich (T) and Tn antigens. *Monoclonal Antibody Information, J. Immunol., Meth.* (In Press).

MILNER, J. & METCALFE, S.M. (1982). A new monoclonal antibody which agglutinates erythrocytes from cancer patients. *Lancet*, **ii**, 1100.

SPRINGER, G.F. & DESAI, P.R. (1975). Human blood-group MN and precursor specificities: structural and biological aspects. *Carbohydr. Res.*, **40**, 183.

SPRINGER, G.F., DESAI, P.R. & BANATWALA, I. (1975). Blood group MN antigens and precursors in normal and malignant human breast glandular tissue. *J. Natl. Cancer Inst.*, **54**, 335.